# Supplementary material for: Network coevolution drives segregation and enhances Pareto optimal equilibrium selection in coordination games
Source: Sci Rep. 2023 Feb 17;13:2866. doi: 10.1038/s41598-023-30011-5 (PMC9938167; doi:10.1038/s41598-023-30011-5)
Supplement: Supplementary file 1 — Supplementary Information. [file 41598_2023_30011_MOESM1_ESM.pdf]

Supplementary Information on ”Network coevolution drives segregation and enhances Pareto optimal equilibrium selection in coordination games”

Miguel A. González Casado[1,3], Angel Sánchez[1,2], and Maxi San Miguel[3]

[1]Grupo Interdisciplinar de Sistemas Complejos (GISC), Universidad Carlos III de Madrid, 28911 Leganés, Spain

[2]Instituto de Biocomputación y Física de Sistemas Complejos (BIFI), Universidad de Zaragoza, 50018 Zaragoza, Spain

[3]Institute for Cross-Disciplinary Physics and Complex Systems IFISC (CSIC-UIB), Campus UIB, 07122 Palma de Mallorca, Spain

S1 Previous Results on Coordination Games

In this first section we present the main results we reproduced that we use as our point of departure in the paper's main body. With respect to Pure Coordination Games, we compute the dependence of α and the density of active links ρ (see Methods for the variables' definitions) on the mean degree $\langle k \rangle$ of the network considered, when the game is played in an Erdös-Rényi network, for both update rules. In Fig. S1 we depict the results obtained for α and ρ as a function of $\langle k \rangle$ for the RD and UI updates rules.

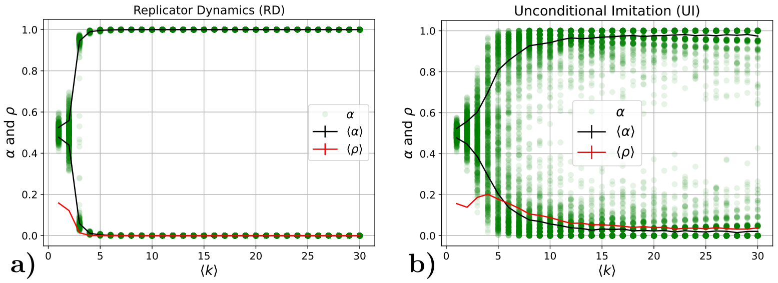

Figure S1: Results for α (single realizations in green and average value in black) and ρ (red) in the frozen state for a) the RD update rule and b) the UI update rule when the PCG is played in an Erdös-Rényi network. We average separately the values for $\alpha > 0.5$ and $\alpha < 0.5$ to account for coordination on action A and B, respectively. Results are averaged over 500 realizations.

Above all, with these plots we want to answer the following question: *Is the system able to reach full coordination?* Let us focus on the cases of large $\langle k \rangle$, since these are the ones we will focus on in this work. A deeper explanation covering all values of $\langle k \rangle$ can be found on the original paper. For large values of $\langle k \rangle$, update rules completely determine the ability of the system to reach full coordination. If we analyze first the RD case, we see that for $\langle k \rangle = 4$ we have almost $\rho \sim 0$ and $\alpha \sim 0$ or 1, meaning that the system is able to perfectly reach full coordination for $\langle k \rangle \geq 4$. For small $\langle k \rangle$ there are still cases in which the system freezes in a configuration which is not fully coordinated, but these cases are a minority and disappear when we go for larger values of $\langle k \rangle$. For the UI case, nonetheless, even for large $\langle k \rangle$ there are a lot of realizations in which the system reaches a frozen configuration far from full coordination. Although as we increase $\langle k \rangle$ the number of realizations ending in full coordination increases, we always have some frozen configurations contrary to what we obtained for the RD case. This result is expected due to the microscopic dynamics of the Unconditional Imitation rule. In the end, if you always imitate the best performing neighbor, there are plenty of possible configurations

in which this rule leads to the coexistence between nodes using different actions. Finally, it is worth mentioning that, since both actions are completely equivalent, the distribution of α is completely symmetric with respect to $\alpha = 0.5$. By and large, we see that these results are in agreement with our main reference (see their Fig. 1).

Now, let us focus on General Coordination Games. We want to know which equilibrium is selected in the absence of coevolution for both update rules. To do so, we set up an Erdös-Rényi network with a fixed value $\langle k \rangle$ for which we obtained that the system is, in principle, able to reach full coordination in a PCG ($\langle k \rangle = 30$ for instance), and we find on which equilibrium the system coordinates depending on the parameters (S, T). Specifically, for each choice (S, T) we measure the average value of α (averaging over 100 realizations), we repeat this process changing (S, T) to cover the whole parameter space and we represent this average value of α in the frozen state as a function of (S, T) using different colors (green for A, yellow for B). We present in Fig. S2 the results obtained.

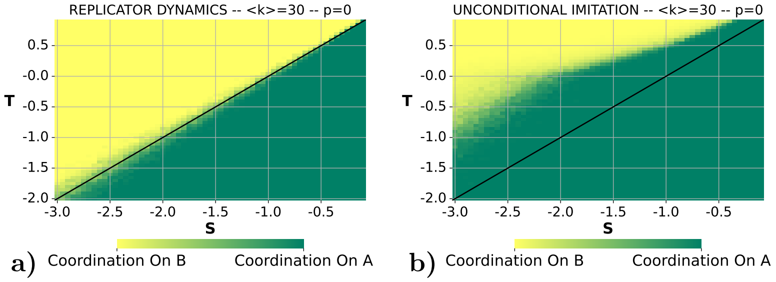

Figure S2: Average value of α for each choice of (S, T) when the GCG played in an E-R network with $\langle k \rangle = 30$ using a) the RD update rule and b) the UI update rule. The granularity in (S, T) is given by the grid. The black line is the *risk-dominant* transition line.

In these Figures we observe that when we use the RD update rule, the system always coordinates on the *risk-dominant* action. This implies that the *green-yellow* transition line (the line dividing the regions of the parameter spade in which the system coordinates on each of the actions) is coincident with the *risk-dominant* transition line. On the other hand, these two lines are not coincident anymore for the UI update rule, so there is an intermediate region in which the system coordinates on the *payoff-dominant* action even if this action is not *risk-dominant.* We see again that these results are in agreement with the results obtained in the reference (see their Fig. 7).

One could wonder "Why is the UI rule much more able to get away from the *risk-dominant* equilibrium?". It comes directly from the fact that, in the UI rule, the agent focuses directly on the best performing neighbor. Thus, close to the *risk-dominant* transition line, it is easier for the agent to choose a neighbor using the *payoff-dominant* action. Basically, close to this line, actions are similar enough that a few connections of agents using the *payoff-dominant* action compensate for their connection with agents using the *risk-dominant* one. All in all, in the RD rule, the agent chooses one neighbor randomly, and it may copy their strategy or not. On the other hand, in the UI rule, the agent directly copies the best performing neighbor, and in a random initial configuration in which both actions are sufficiently similar, the mechanism of the UI rule is going to reinforce any agent with a slightly larger number of neighbors if both are using the *payoff-dominant* action, and once this happens, the *payoff-dominant* action is able to take over.

S2 Defrost of Frozen Configurations

In the paper's main body we mentioned that, for the UI rule, for which some specific realizations freeze in an uncoordinated configuration in the absence of coevolution, we could wonder if coevolution helps unfreeze these specific realizations, helping the system to reach full coordination without fragmenting it. We specified that we analyzed if this was the case, but the change in behavior was not significant. To check it we depict first in panel a) of Fig. S3 the variable ψ that we define as the proportion of realizations ending in full coordination as a function of the mean degree $\langle k \rangle$ of the network. First, it is direct to test with this plot that ψ grows with $\langle k \rangle$, reaching $\psi = 0.79(1)$ for $\langle k \rangle = 30$. Now, in panel b) of the same Figure, we take $\langle k \rangle = 30$ and we add coevolution, computing ψ as a function of the rewiring probability p. In this plot we present as well a black line at $\psi = 0.79$ corresponding to the case without coevolution. In this plot we obtain that for small values of p, ψ is indeed above 0.79. For instance, for $p = 0.11$, $\psi = 0.82(1)$. So, with this result we confirm that a small value of the rewiring can help unfreeze uncoordinated realizations, although this effect is marginal. Thus, we can conclude that, for small values of p, realizations in which the system reached full coordination in the absence of coevolution continue doing so, and most realizations ending in a frozen configuration now fragment into two pieces, although a marginal proportion of them unfreeze and reach full coordination.

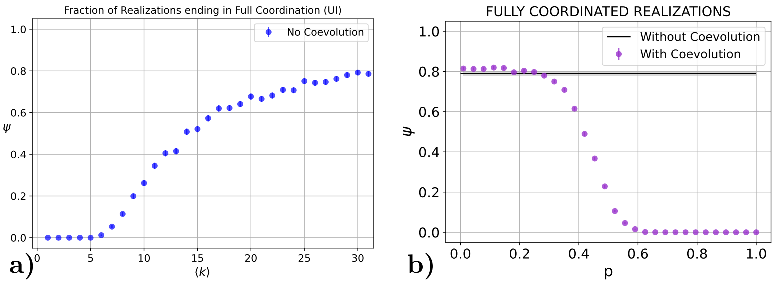

Figure S3: a) Proportion of realizations ending in full coordination as a function of the mean degree of the network, for a Pure Coordination Game without coevolution using the Unconditional Imitation rule. b) Proportion of realizations ending in full coordination as a function of the rewiring probability p for fixed $\langle k \rangle = 30$, for a Pure Coordination Game using the Unconditional Imitation rule.

S3 Probability Distributions: Size and Number of Fragments

In the paper's main body we presented in two plots the average value of the size and the number of fragments as a function of p. In these same plots, single realizations were depicted in semi-transparent points such in order to mimic the probability distributions as if they were "seen from above". We present here an example of the actual probability distributions (the ones for the Unconditional Imitation rule in a Pure Coordination Game) for each choice of p, to help the reader visualize them and get familiar with the Figures of the paper's main body.

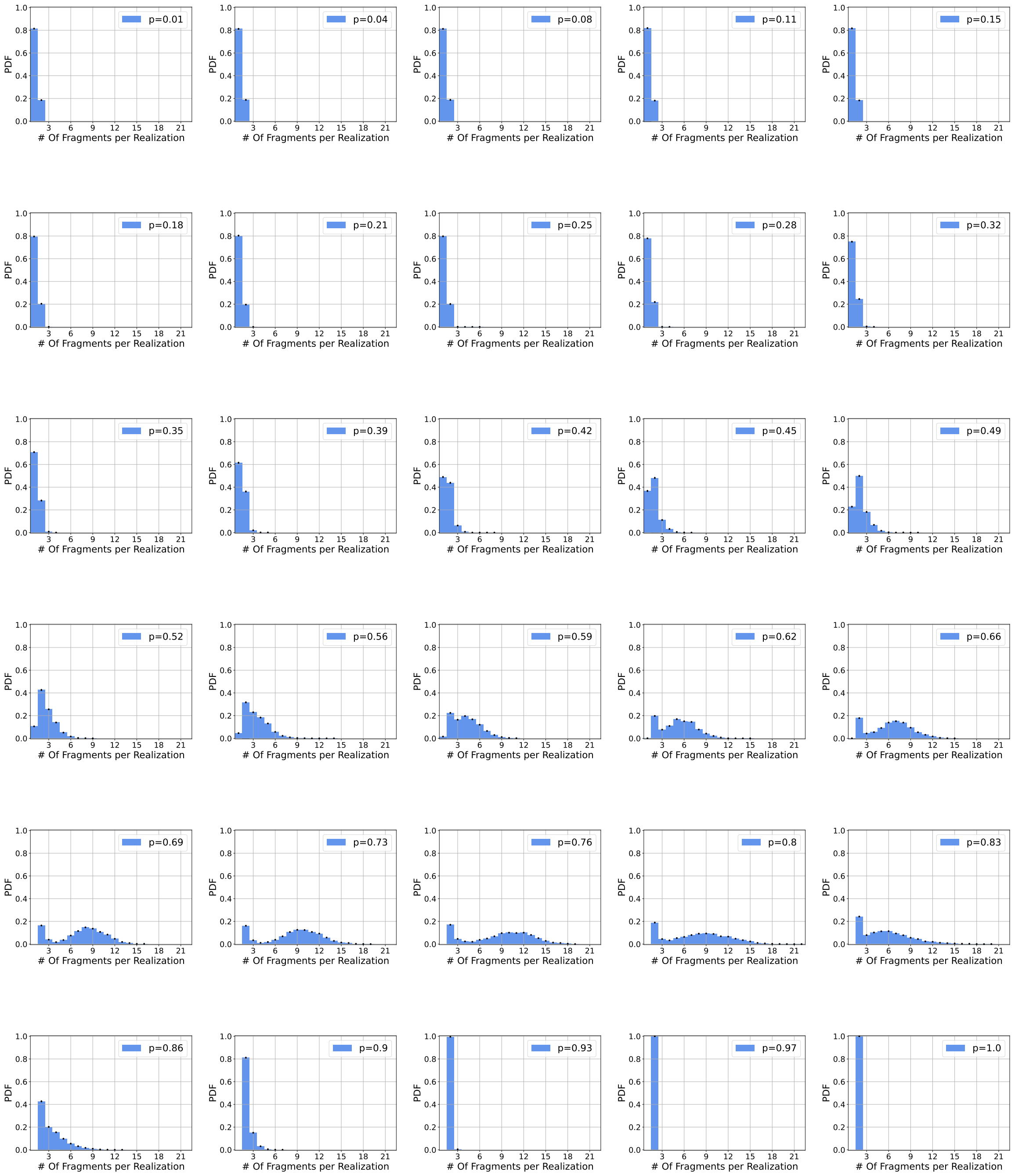

Figure S4: Probability Distribution Functions of the number of fragments for each value of p considered. (Case PCG - UI, $\langle k \rangle = 30$).

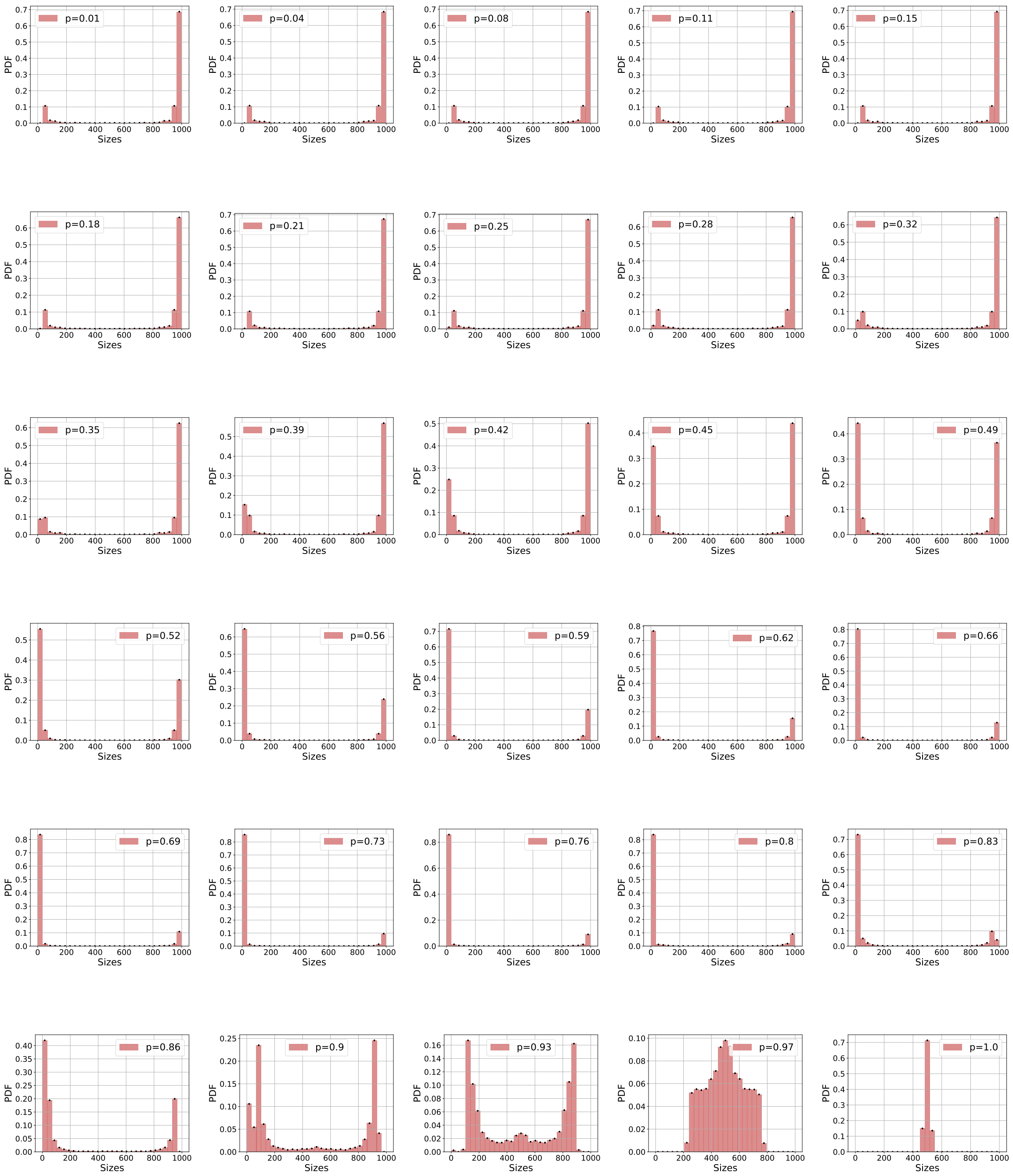

Figure S5: Probability Distribution Functions of the size of the fragments for each value of p considered. (Case PCG - UI, $\langle k \rangle = 30$).

S4 Fragmentation Transition in a General Coordination Game

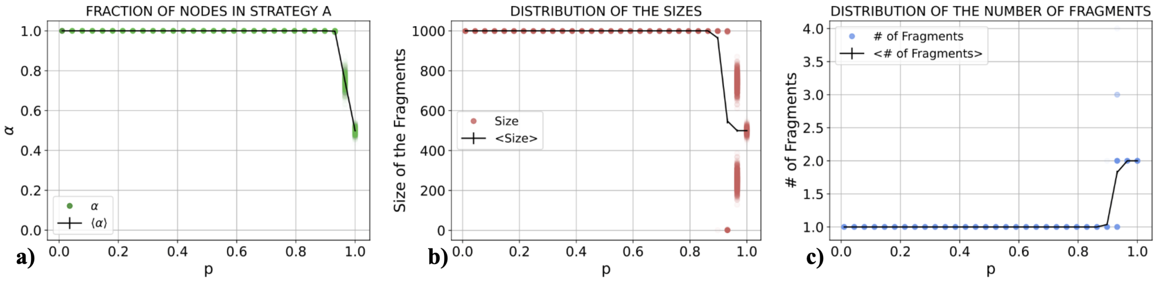

Figure S6: Results for α, the size and the number of fragments (single realizations in green, red and blue, respectively, and average values in black) as a function of p for a GCG played in an E-R network with $\langle k \rangle = 30$, using the RD update rule with parameters $(S, T) = (-1.5, -1.5)$.

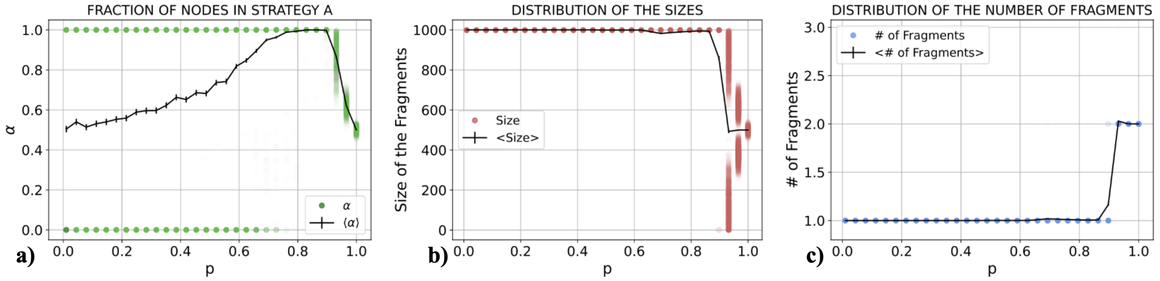

Figure S7: Results for α, the size and the number of fragments (single realizations in green, red and blue, respectively, and average values in black) as a function of p for a GCG played in an E-R network with $\langle k \rangle = 30$, using the RD update rule with parameters $(S, T) = (-2, -1)$.

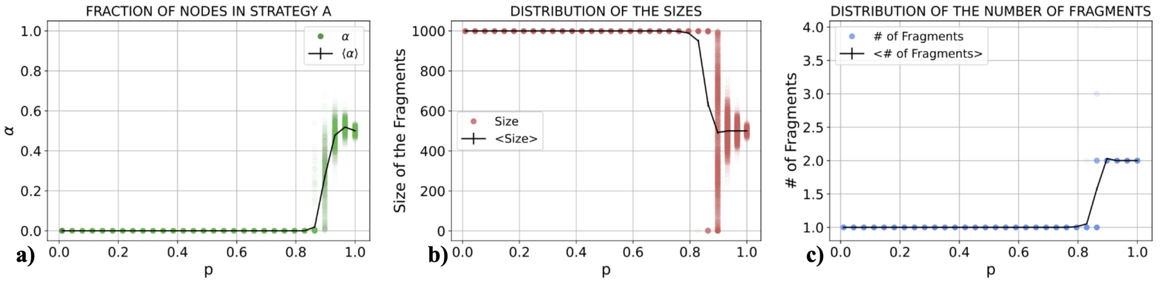

Figure S8: Results for a) α, b) the size and c) the number of fragments (single realizations in green, red and blue, respectively, and average values in black) as a function of p for a GCG played in an E-R network with $\langle k \rangle = 30$, using the RD update rule with parameters $(S, T) = (-2.5, -0.5)$.

In the paper's main body we included simply panels a) of Figs. S6, S7 and S8 for the Replicator Dynamics rule, arguing that the other two plots (sizes and number of fragments) displayed a similar behavior to the one observed for Pure Coordination Games, and hence it was not necessary to include them again for the case of General Coordination Games. We present here these three Figures including them to check that indeed the nature of the transition is similar to the one observed for PCG. Furthermore, we include as well (Figs. S9, S10 and S11) the same plots for the Unconditional Imitation

rule, although it was excluded from the paper's main body to simplify the explanation. It is direct to check first that the fragmentation transition has the same nature as well to the one observed for PCG, and second that if we take (S, T) sufficiently close to the transition line, we have as well for the UI case a transition to a regime in which the system fully coordinates on the *payoff-dominant* action even if this action is not *risk-dominant*.

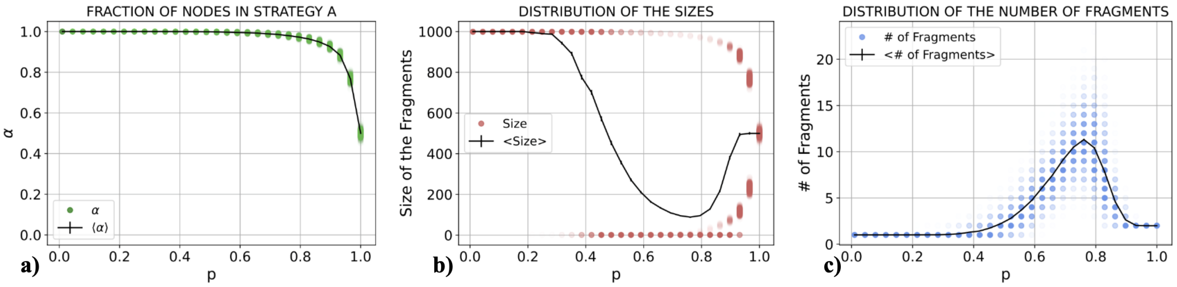

Figure S9: Results for a) α, b) the size and c) the number of fragments (single realizations in green, red and blue, respectively, and average values in black) as a function of p for a GCG played in an E-R network with $\langle k \rangle = 30$, using the UI update rule with parameters $(S, T) = (-2, -1)$.

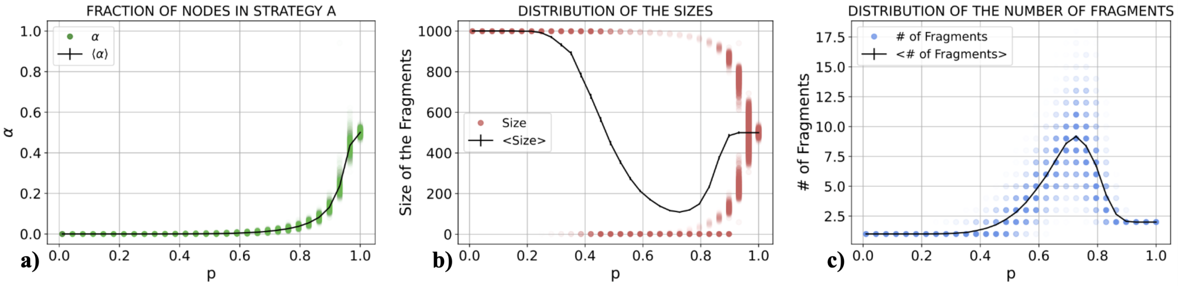

Figure S10: Results for a) α, b) the size and c) the number of fragments (single realizations in green, red and blue, respectively, and average values in black) as a function of p for a GCG played in an E-R network with $\langle k \rangle = 30$, using the UI update rule with parameters $(S, T) = (-4, +1)$.

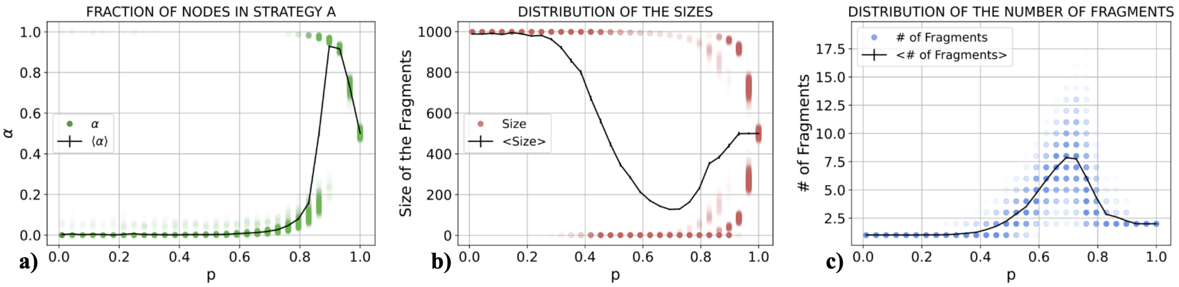

Figure S11: Results for a) α, b) the size and c) the number of fragments (single realizations in green, red and blue, respectively, and average values in black) as a function of p for a GCG played in an E-R network with $\langle k \rangle = 30$, using the UI update rule with parameters $(S, T) = (-1.5, 0.85)$.

S5 Evolution of the Transition Line (UI rule)

Finally, we present the plot showing the evolution of the *green-yellow* transition line with the rewiring probability p for the Unconditional Imitation update rule. Indeed, we observe how this line displaces towards the region in which action B is *risk-dominant*, widening the region in which the system is able to coordinate on the *payoff-dominant* action even if this action is not *risk-dominant*, similar to what we observed for the Replicator Dynamics rule.

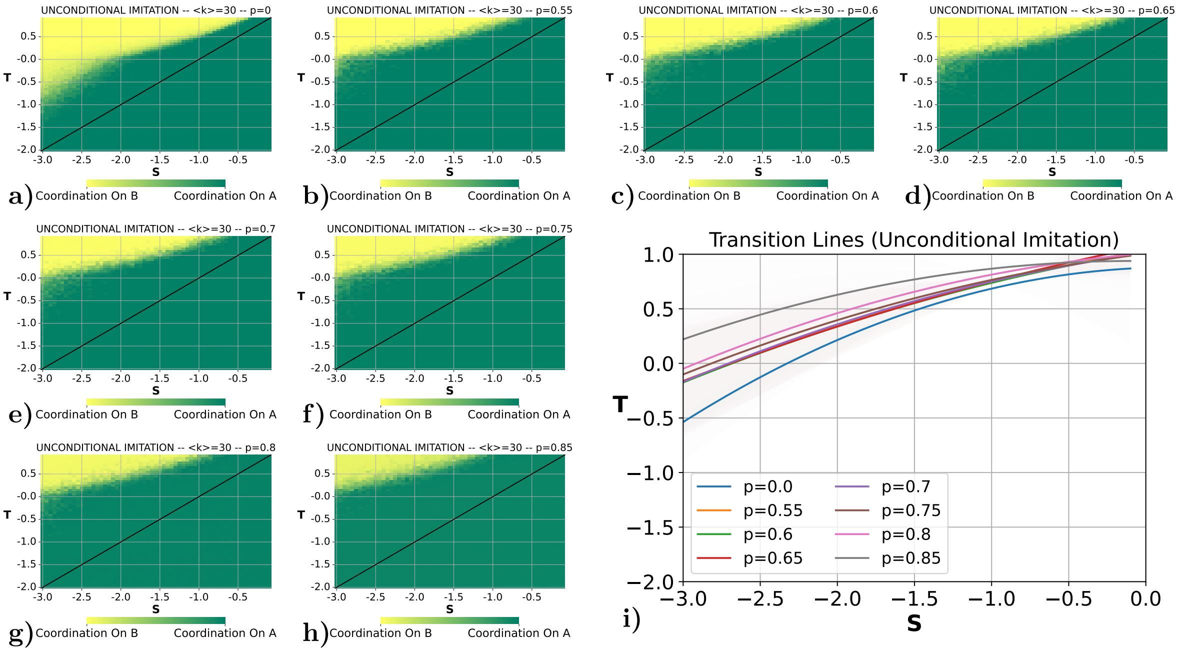

Figure S12: **a)-h) Panels:** (S, T) diagrams showing the average value of α for different choices of p. **h) Panel:** Summary of the *green-yellow* transition lines obtained for different choices of p.

S6 Cases $\langle k \rangle = 10,\ 20$

Replicator Dynamics

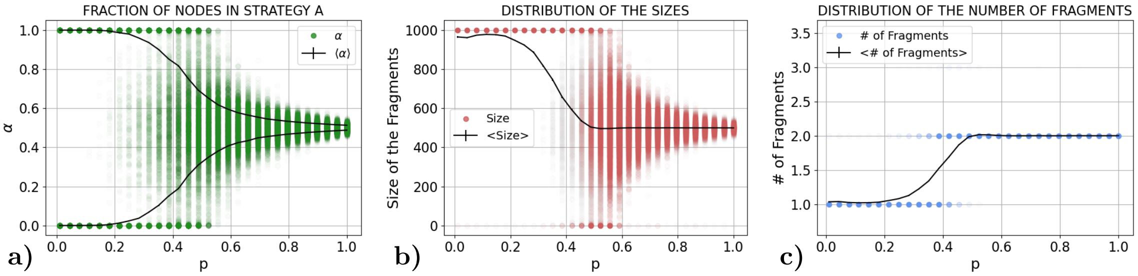

Figure S13: Results for a) α, b) the size and c) the number of fragments (single realizations in green, red and blue, respectively, and average values in black) as a function of p for a PCG played in an E-R network with $\langle k \rangle = 10$ using the RD update rule.

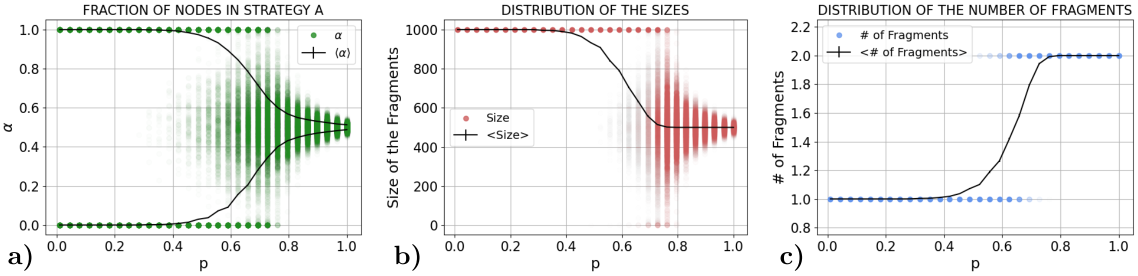

Figure S14: Results for a) α, b) the size and c) the number of fragments (single realizations in green, red and blue, respectively, and average values in black) as a function of p for a PCG played in an E-R network with $\langle k \rangle = 20$ using the RD update rule.

Unconditional Imitation

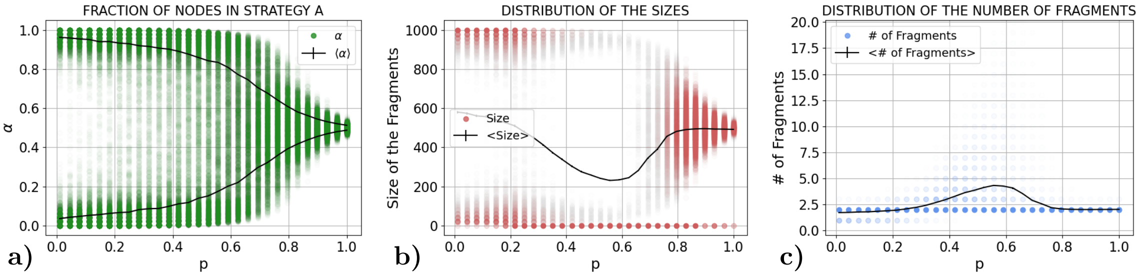

Figure S15: Results for a) α, b) the size and c) the number of fragments (single realizations in green, red and blue, respectively, and average values in black) as a function of p for a PCG played in an E-R network with $\langle k \rangle = 10$ using the UI update rule.

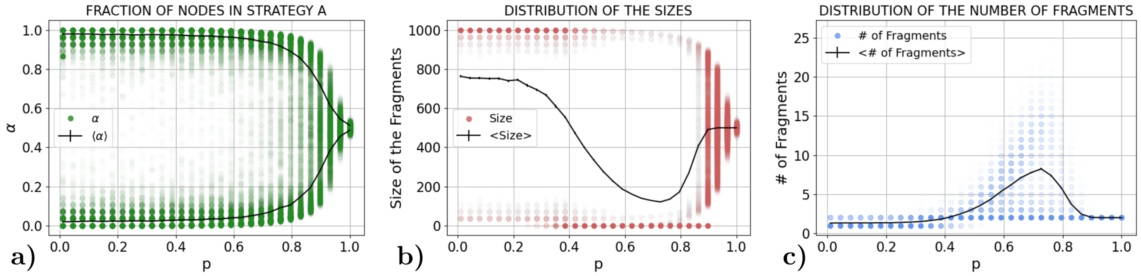

Figure S16: Results for a) α, b) the size and c) the number of fragments (single realizations in green, red and blue, respectively, and average values in black) as a function of p for a PCG played in an E-R network with $\langle k \rangle = 20$ using the UI update rule.
